# Translation, Cross-Cultural Adaptation, and Validation of the Parkinson's Disease Quality of Life Questionnaire (PDQL), the “PDQL-BR”, into Brazilian Portuguese

**DOI:** 10.5402/2011/954787

**Published:** 2011-07-28

**Authors:** Marcos Campos, Carlos Henrique A. de Rezende, Virgilio da C. Farnese, Carlos Henrique M. da Silva, Nívea Macedo de O. Morales, Rogério de Melo C. Pinto

**Affiliations:** Department of Post Graduate Health Sciences, School of Medicine, Federal University of Uberlandia, Avenue Pará No. 1720, 38405-320 Uberlandia, MG, Brazil

## Abstract

Translate, culturally adapt, and validate the “Parkinson's Disease Quality of Life” (PDQL) BR, into Brazilian Portuguese. Fifty-two patients answered the PDQL-BR. Twenty-one patients answered the PDQL-BR again 14 days later. The UPDRS and HY scale was applied. Validation was evaluated using psychometric properties, checking the quality of the data, reliability, and validity. Quality of the data was evaluated based on occurrence of ceiling and floor effects. Reliability was evaluated based on: internal consistency of an item, homogeneity, and reproducibility. Validation was checked through the evaluation of convergent and discriminatory validation. There was no ceiling and floor effect. When evaluating reliability, items 20, 30, and 37 showed correlation of 0.34, 0.26, and 0.37, respectively, to your scale; the other items was higher than 0.4. The alpha Cronbach coefficient was higher than 0.7 for most domains. There was good reproducibility. There were no meaningful changes in the PDQL-BR translation and cross-cultural adaptation.

## 1. Introduction

Parkinson's disease (PD) is a neurodegenerative disease characterized by the occurrence of motor and nonmotor symptoms. Motor symptoms include arrest tremors, stiffness, bradykinesia, and instabilities in posture and gait. Nonmotor symptoms include autonomic dysfunctions that result in changes in blood pressure control and intestinal function. The disease shows an inevitably progressive course and an increasing number of other symptoms. Frequently, Parkinson's disease patients go through periods of depression and dementia. In more advanced stages, complications caused by treatment with dopaminergic drugs result in fluctuations in motor function [1]. The symptoms, comorbidities, and complications of treatment restrict the autonomy and the welfare of affected individuals, leading to prejudices in their professional, social, and emotional lives [2].

In order to evaluate multidimensional features such as those described above, recently special attention has been given to evaluating the quality of life (QL) and, more specifically the perception of welfare in light of the state of the patient health, which has lead to the development of the Health-Related Quality of Life (HRQL) surveys [3]. The development of the HRQL has allowed health care providers to determine a better approach to caring for the patient; the HRQL also places more value on considering the needs and the expectations of the patient and provides a comparison among different groups [3]. The HRQL instruments are divided into two groups: generic and specific. The generic instruments determine general features of health and have advantages, such as wide applicability, that allow them to be used in different groups of patients and allowing comparisons among the groups. The specific surveys may be used in specific situations, such as PD, and their advantage is an approach to specific aspects of the disease being studied, allowing the comparison of individuals in the same group [4].

Four instruments of HRQL have been developed and validated specifically for PD: Parkinson's Disease Questionnaire 39 (PDQ-39), Parkinson's disease quality of life (PDQL), Parkinson's Impact Scale (PIMS), and Parkinson's Lebens Qualitat (PLQ). Among them, the PDQ-39 is considered the most appropriate; however, it has shortcomings in the evaluation of self-image sleep disturbance and sexuality of patients. Because the PDQL takes into account these problems, it has become an alternative to PDQ-39 [5]. For these reasons, the PDQL has generated more interest, and there has been an increase in translation and validations for several languages and cultures [5].

The simple translation of HRQL instruments into the language of countries or cultures different from the ones in which they were developed is insufficient, because the influence of culture and linguistics may necessitate conceptual changes [6, 7]. While there is no universal agreement about how these instruments must be translated and culturally adapted, back translation is thought to be the most appropriate mechanism. After translation and cross-cultural adaptation, the instrument should be validated in order to determine if the translation process changed the psychometric properties of the original instrument [7]. The aim of this study was to translate, culturally adapt, and validate PDQL into Brazilian Portuguese.

## 2. Materials and Methods

### 2.1. Ethics

The study was approved by the Ethics in Research Committee of the Federal University of Uberlandia (UFU). All of the patients agreed to participate in the study (consent term). The use of the PDQL is in accordance with the norms of use of MAPI Trust Research [8].

### 2.2. Instrument

Following the international recommendations for translation and cross-cultural adaptation [6–8], the original instrument was initially translated into Brazilian Portuguese by two English language teachers that were born in Brazil and are fluent in the English language. Both versions were compared by a neurology professor who is a Brazilian native and who consolidated the first version. This version was then translated into the original language (backtranslation) [7] by an English language teacher from the United Kingdom who is fluent in Brazilian Portuguese. This version was compared to the original version before being accepted for cultural adaptation. For cultural adaptation, the instrument was applied given to a group of patients with PD in order to consolidate the final version to be validated, the PDQL-BR. 

The PDQL-BR, like the original, is composed of thirty-seven items divided into four domains: parkinsonian symptoms (14 items), systemic symptoms (9 items), emotional function (7 items), and social function (7 items). Each item is scored from 1 to 5, and the total score of the instrument is given by the sum of the averages of the scores from each domain. Higher scores represent a better perception of HRQL by the patient [9].

### 2.3. Patients

A convenience sample, composed of patients diagnosed with PD according to the UK Parkinson's Disease Society brain bank criteria, came from the movement disturbances ambulatory clinic of the UFU, the association of patients with parkinson's disease in Uberlandia, and private neurology clinics. Patients that were unable to participate in everyday activities due to comorbidities other than PD and patients with serious cognitive damage who scored less than eighteen points on the Miniexam for mental state [10] were excluded from the study.

### 2.4. Procedures

All of the patients went through a clinical evaluation by a neurologist. The disease was staged according to the modified classification of Hoehn and Yahr (HY) [11] and then patients were classified into phases. The initial phase was composed of HY stages 1.0, 1.5, and 2.0, the moderate phase HY stage 2.5, and the advanced phase HY stages 3.0, 4.0, and 5.0. A global evaluation of functional damage was done for each patient using the Unified Parkinson's Disease Rating Scale (UPDRS) [12]. After clinical evaluation, the patients answered a social-demographic questionnaire by interview, the PDQL-BR and the Beck Depression Inventory (BDI) [13], and were invited to return within 14 days to answer the PDQL-BR again.

### 2.5. Validation

The PDQL-BR was validated by checking the psychometric properties of the instrument and the quality of the data; reliability and validity were analyzed. The quality of the data was verified by checking the occurrence of floor and ceiling effects and the percentage of lost data. The presence of floor or ceiling effects indicates that the instrument has difficulty detecting differences in the perception of welfare among individuals with the lowest possible score (floor effect) or with the highest possible score (ceiling effect). Lost data are data which could not be used in the evaluation and interpretation of the application of the instrument, and a loss of less than 10% of the data obtained was considered reasonable [14].

The reliability of the instrument was evaluated by determining the internal consistency of the item, the reliability of the internal consistency, and reproducibility. The internal consistency of an item evaluates the ability of each item to contribute to the creation of a basis for the scale that it represents [15]. It was determined by correlating each item to its domain and evaluating the percentage of items that correlate in a satisfactory way to their domain (success rate) [14]. The reliability of the internal consistency or homogeneity of data of a domain or scale is the extent to which all of their data define different aspects of the same attribute [15]. The reliability of internal consistency is guaranteed when the items are moderately correlated to each other and when each item correlates to the total score [15]. Reliability was calculated for each domain and for the PDQL-BR. Reproducibility is the capacity of the instrument, in stable conditions, to reproduce the same results obtained from an initial evaluation [15]. Reapplication of PDQL-BR occurred within a period of 14 days after the first test.

Validity determines if the tool really measures the concepts it is supposed to measure, and that it does not measure what it isn't intended to measure [16]. Discriminatory validity and convergent validity were studied. Discriminatory validity verifies the capacity of the instrument to discriminate among subgroups of patients in different clinical states [9] and was determined by comparing the PDQL-BR scores and the parkinsonian symptoms domain and the systemic symptoms among the groups of patients in initial, moderate, and advanced phases of the disease. Convergent validity determines the amount of association between two measures of the same construct [9]. It was determined by correlating scores from the parkinsonian symptoms domain of the PDQL-BR and scores of the UPDRS III and among emotional function scores from the PDQL-BR and UPDRS I and the BDI scores.

### 2.6. Statistics

Descriptive statistics were used for the characterization of the sample. For correlation tests, the Spearman correlation coefficient was used because it is considered a satisfactory correlation when it is over 0.4. For the evaluation of internal consistency of an item, the success rate was considered satisfactory when it was greater than 80%. For the reliability evaluation of internal consistency, the alpha-Cronbach coefficient was used, and values over 0.7 were considered satisfactory internal consistency for comparison among groups. For the evaluation of reproducibility of the instrument and discriminatory validity, the Mann-Whitney test was used. The level considered significant was *P* < 0.05.

## 3. Results

### 3.1. Instrument

During the process of cross-cultural adaptation, item 20 of the scale referred to “*períodos de liga/desliga*” was modified to “*períodos de trava/destrava* (*momentos com/sem ação dos remédios*)” to improve the understanding of the phase.

### 3.2. Patients

Fifty-eight patients were invited to participate in the study. Five were excluded because they had cognitive damage, and one did not fit the criteria for diagnosis of PD. Thirty-six (69.2%) were male. The patients' ages varied from 37 to 88 years (average: 64.83 PD 11.81). Forty-five (86.5%) of the patients were Caucasian, and 33 (63.5%) were married. The clinical characteristics are presented in Table 1.

### 3.3. Validation

#### 3.3.1. Quality of Data

Floor and ceiling effects were not observed for any of the PDQLBR domains, and the rate of lost data varied among domains from 0.3 to 0.6%.

#### 3.3.2. Reliability

For internal consistency of items, all of the items in both the systemic symptoms and social function domains had correlations greater than 0.4 with their respective scales (success rate of 100%). In the parkinsonian symptoms domain, the items 20 and 30 had correlations of 0.34 and 0.26, respectively, but the other items had correlations that were greater than 0.4 (success rate of 85.71%). In the emotional function domain, item 37 had a correlation of 0.37. The other items had correlations that were greater than 0.4 (success rate of 88.89%). For evaluation of the reliability of internal consistency, the alpha Cronbach coefficient of the tool and for each domain was higher than 0.65 and can be seen in Table 2. Twenty-one patients answered the PDQLBR again for the evaluation of reproducibility. The second occurrence was fourteen days after the first application of the instrument. There were no statistically significant differences between the average scores of the two PDQL-BR applications.

#### 3.3.3. Validity

In the evaluation of discriminatory validity of the PDQL-BR, the parkinsonian symptoms and the systemic symptoms scores showed differences among all the phases of the disease because the average scores of the patients in each phase differed, with lower scores as the disease advanced. For convergent validity, correlation between the parkinsonian symptoms domain of the PDQL-BR and UPDRS III was 0.78. For the emotional function domain of the PDQL-BR, correlations with the UPDRS I and the DBI were 0.59 and 0.57, respectively, with significance level of 0.01.

## 4. Discussion

The clinical characteristics of the evaluated patients were similar to the characteristics observed in other studies of translations and validation of PDQL. The population of the study was similar with regard to the evolution of the disease [17–19]. The data obtained were satisfactory for analysis. The data collection by interview minimized the loss of data, and the absence of floor or ceiling effects indicated that, according to theory, the translated version of the instrument kept its capacity to determine changes. It is possible that the patients did not have a good understanding of item 20 “*períodos de trava/destrava* (*momentos com/sem ação dos remédios*),” because this item had been translated as “períodos liga/desliga” in the translation process and then had generated incapacity of understanding by the patients. Even after a change in the translation, it is possible that patients still did not fully understand the symptom, causing low correlation with the domain that it represents. However, the low correlations of items 30 “movimentos repentinos e não controlados” and 37 “*sente-se preocupado (a) com as possíveis consequências de uma operação por causa de sua doença*” are possibly associated with the inner characteristics of the studied population. In the first item, patients from nonspecialized ambulatory clinics before the inclusion of the disturbance movement ambulatory clinic of the Federal University of Uberlandia were medicated with low doses of levodopa and, consequently, had low frequencies of motor fluctuation, the symptom referred to in item 30. However, they indicated that they had significantly damage from the disease due to the use of very low doses of levodopa. Item 37 does not depend on the clinical condition of the patient. Surgical procedures for treatment of PD are not routinely performed at the clinics from which the patients were recruited, which may not mean anything for most patients. The instrument showed itself to be reliable, because the alpha Cronbach coefficient for most domains was higher than 0.7. Only in the systemic symptoms domain was it 0.65. However, great correlation was observed among the principal studies of validation of the instrument in different cultures, according to Table 2.

The translated version of the instrument demonstrated good reproducibility because there were no significant variations in the scores obtained from the first and the second application. For the validity evaluation, we observed that the instrument had a good convergent validity and showed good association between the parkinsonian symptoms domain and the UPDRS III motor evaluation and also among the emotional function domain and the evaluation for UPDRS I and BDI scores. We also observed that the tool had a good capacity to discriminate among patients in the initial, moderate, and advanced stages.

## 5. Conclusions

We conclude that the translated version of PDQL (PDQL-BR) did not undergo meaningful changes in the process of translation and cross-cultural adaptation and keeps its psychometric proprieties well preserved. A careful interpretation of the evaluation of answers to items 20, 30, and 37 is recommended.

The current study showed that PDQL-BR is a valid instrument for use in Brazil; however, the validation process is dynamic and goes through changes over time. Only frequent use of the instrument will result in more consistent theoretical evaluations. Thus, we understand that more studies using the instrument will be fundamentally important for increasing the knowledge of it.

## Figures and Tables

**Table 1:** Clinical characteristics of patients.

Clinical characteristics	(*N*)	(%)
Duration of illness		
Up to 5 years	25	48.1
From 6 to 10 years	13	25.0
More than 10 years	13	25.0
Unknown	1	1.9
Staging		
Initial phase (HY 1/1.5/2)	8	15.3
Moderate phase (HY 2.5)	23	44.2
Advanced phase (HY 3/4/5)	21	40.4

**Table 2:** Comparison of the alpha-Cronbach coefficients among studies.

	De Boer et al. [9]	Hobson et al. [2]	Serrano-Dueñas et al. [19]	Present study
PDQL	0.94	0.95	0.92	0.93
Parkinsonian symptoms	0.86	0.87	0.85	0.83
Systemic symptoms	0.80	0.77	0.69	0.65
Emotional function	0.87	0.87	0.78	0.79
Social function	0.82	0.85	0.81	0.80
